# The antibody response induced FMDV vaccines in sheep correlates with early transcriptomic responses in blood

**DOI:** 10.1038/s41541-019-0151-3

**Published:** 2020-01-03

**Authors:** Luc Jouneau, David J. Lefebvre, Fleur Costa, Aurore Romey, Sandra Blaise-Boisseau, Anthony Relmy, Yan Jaszczyszyn, Cloelia Dard-Dascot, Sébastien Déjean, Nicolas Versillé, Edouard Guitton, Pascal Hudelet, Marianne Curet, Kris De Clercq, Labib Bakkali-Kassimi, Stéphan Zientara, Bernard Klonjkowski, Isabelle Schwartz-Cornil

**Affiliations:** 1 Université Paris-Saclay, INRA, VIM, Domaine de Vilvert, 78350 Jouy-en-Josas, France; 2 Sciensano, Scientific Direction of Infectious Diseases in Animals, Service for Exotic Viruses and Particular Diseases, Groeselenberg 99, 1180 Brussels, Belgium; 3 Université Paris-Est, ANSES, Ecole Nationale Vétérinaire d’Alfort, INRA, Laboratoire de santé animale, UMR Virologie, Maisons-Alfort, France; 4 Université Paris-Saclay, Université Paris-Sud, CNRS, CEA, Institute for Integrative Biology of the Cell (I2BC), Gif-sur-Yvette, France; 5 Université de Toulouse, Université Paul Sabatier, CNRS, Institut de Mathématiques de Toulouse, UMR5219, 31062 Toulouse Cedex, France; 6 SEPPIC Paris La Défense, Paris, France; 7 INRA, Plate-Forme d’Infectiologie Expérimentale (PFIE), UE1277, 37380 Nouzilly, France; 8 Merial S.A.S., 29 Avenue Tony Garnier, 69007 Lyon, France

**Keywords:** Adjuvants, Live attenuated vaccines, Inactivated vaccines, Viral infection

## Abstract

Foot and mouth disease (FMD) is a highly contagious viral disease with high economic impact, representing a major threat for cloven-hooved mammals worldwide. Vaccines based on adjuvanted inactivated virus (iFMDV) induce effective protective immunity implicating antibody (Ab) responses. To reduce the biosafety constraints of the manufacturing process, a non-replicative human adenovirus type 5 vector encoding FMDV antigens (Ad5-FMDV) has been developed. Here we compared the immunogenicity of iFMDV and Ad5-FMDV with and without the ISA206VG emulsion-type adjuvant in sheep. Contrasted Ab responses were obtained: iFMDV induced the highest Ab levels, Ad5-FMDV the lowest ones, and ISA206VG increased the Ad5-FMDV-induced Ab responses to protective levels. Each vaccine generated heterogeneous Ab responses, with high and low responders, the latter being considered as obstacles to vaccine effectiveness. A transcriptomic study on total blood responses at 24 h post-vaccination revealed several blood gene module activities correlating with long-term Ab responses. Downmodulation of T cell modules’ activities correlated with high responses to iFMDV and to Ad5-FMDV+ISA206VG vaccines as also found in other systems vaccinology studies in humans and sheep. The impact of cell cycle activity depended on the vaccine types, as it positively correlated with higher responses to iFMDV but negatively to non-adjuvanted Ad5-FMDV. Finally an elevated B cell activity at 24 h correlated with high Ab responses to the Ad5-FMDV+ISA206VG vaccine. This study provides insights into the early mechanisms driving the Ab response induced by different vaccine regimens including Ad5 vectors and points to T cell modules as early biomarker candidates of different vaccine-type efficacy across species.

## Foot and mouth disease virus: correlates of protection

Foot and mouth disease virus (FMDV) is a serious pathogen of cloven hoofed mammals and is of high economic and veterinary importance. Inactivated vaccine (iFMDV) is effective but difficult to produce because of high biosafety level requirements; non-replicating adenovirus vectors carrying key FMDV antigens (Ad5-FMDV) might therefore represent an attractive alternative. Isabelle Schwartz-Cornil and colleagues use sheep to systematically compare vaccination with adjuvanted iFMDV, adjuvanted Ad5-FMDV, or non-adjuvanted Ad5-FMDV. All vaccines produce neutralizing antibody responses which are stable to at least one year, however the iFMDV group elicits the strongest response, followed by the adjuvanted Ad5-FMDV. Ad5-FMDV alone produces weak antibody titers. Blood transcriptomic analysis performed in the first 24 h following vaccination identifies a reduced T cell gene expression module as a correlate of high neutralizing antibody titers. Blood gene expression might therefore offer insights into the mechanistic underpinnings of humoral immunity as well as provide useful biomarker correlates of protection.

## Introduction

The foot and mouth disease virus (FMDV) is a member of the Picornaviridae family, genus *Aphthovirus*, and is responsible of a contagious vesicular disease affecting cloven-hooved mammals, which has a major impact on animal productions and trade.^1^ FMDV is currently endemic in Asia, Africa, and Venezuela in South America and causes severe economic consequences.^2^ Due to its contagiousness, FMDV is under very strict regulatory control by the World Organization for Animal Health (OIE) which limits the trade of susceptible animals and their products from endemic countries.^3^ In FMDV-free zones such as countries from the European Union, FMDV outbreaks have major economic consequences due to implementation of control measures that involve susceptible animal depopulations and vaccination, and to losses in animal products exportations.^4^ FMDV is genetically unstable and exists in seven distinct serotypes with many subtypes that induce limited cross-protection. Vaccination with inactivated FMDV (iFMDV) against the circulating serotypes, formulated in aqueous aluminum hydroxide and saponins or oil-based adjuvants, is an effective control measure.^5^ Semi-annual mass vaccination is practiced in endemic countries and ring vaccination around outbreak areas has been conducted in non-endemic countries to complement the depopulation strategy.^4^ iFMDV vaccine production requires handling of live FMDV in high containment facilities, is costly and raises safety issues. Therefore recombinant vaccines, such as human replication-defective adenovirus 5 vector encoding capsid and capsid-processing proteins (Ad5-FMDV), have been successfully developed^6,7^ and have been conditionally authorized by the United States Department of Agriculture (USDA) in case of accidental or intentional outbreaks in the USA. Pig and cattle vaccinated with different versions of Ad5-FMDV showed partial clinical protection against an FMDV challenge, with induction of neutralizing antibodies (Abs), which are considered as key protection effectors, in a fraction of the vaccinated animals.^8–10^ Importantly, in order to increase the vaccine potency and decrease dosages, Ad5-FMDV has been associated with adjuvants such as poly IC^11^ or a lipid polymer which induced higher levels of immunogenicity and full protection in cattle.^8^ Ad5-FMDV vaccines offer the advantage to be manufactured in BSL2 containment, without the need to produce infectious FMDV batches.^12,13^ Ad5-FMDV therefore stands as a very promising control tool for use against the FMDV global threat and especially in case of outbreak in non-endemic countries.

Depending on susceptible species, the pathogenicity of FMDV is variable: cattle and pigs present the highest severity of clinical signs, whereas sheep and goat show a more discrete disease and can be important in disease dissemination.^14^ Furthermore, outbreaks of FMD within and around the European Union member states have involved sheep.^14^ To the best of our knowledge, the immunogenicity of Ad5-FMDV has not been evaluated in sheep, and it is not known how it compares to iFMDV immunogenicity in that species, also taking into account that sheep appear to develop different serological responses to iFMDV vaccines as compared to cattle and other ruminants, depending on FMDV serotypes.^14^

In this work, we analyzed in sheep the humoral responses induced by a benchmark iFMDV vaccine of the O1 Manisa serotype formulated in aluminum hydroxide plus saponin and the ones induced by a non-replicative Ad5-FMDV version, which comprises P1, 2A, and 2B of serotype O1 Manisa fused in frame with the non-structural proteins 3B (partial) and 3C of the serotype A12, which we administered alone or with a water-in-oil-in-water emulsion, Montanide ISA206VG, an efficient adjuvant for sheep and cattle.^14^ The three vaccines induced different ranges of serological responses, the highest with iFMDV and the lowest with Ad5-FMDV, and all induced heterogeneous levels of responses, especially in the Ad5-FMDV vaccinated groups. As poor responders represent a bottleneck for vaccine effectiveness, we searched for mechanisms associated with the Ab response magnitude, by exploiting correlations with the early transcriptomic responses to the vaccines. Indeed, in the last years, the analysis of the early transcriptomic response to vaccines in the blood of human patients have been used to shed light on the mechanisms involved in effective vaccine response,^15–19^ and recently, it was successfully used in sheep.^20^ Regarding Ab response induction, early transcriptomic studies in human mainly involved inactivated or protein vaccines, but not adenovirus-vectored vaccines, to the best of our knowledge. By using blood transcription modules (BTMs) of co-regulated genes through RNA-seq data integration, we identified early gene response signatures in total blood cells which correlated with the response magnitude to each vaccine type.

## Results

### iFMDV, Ad5-FMDV, and Ad5-FMDV+ISA206VG show different potencies to trigger Ab responses with heterogeneity among sheep

**Fig. 1:**
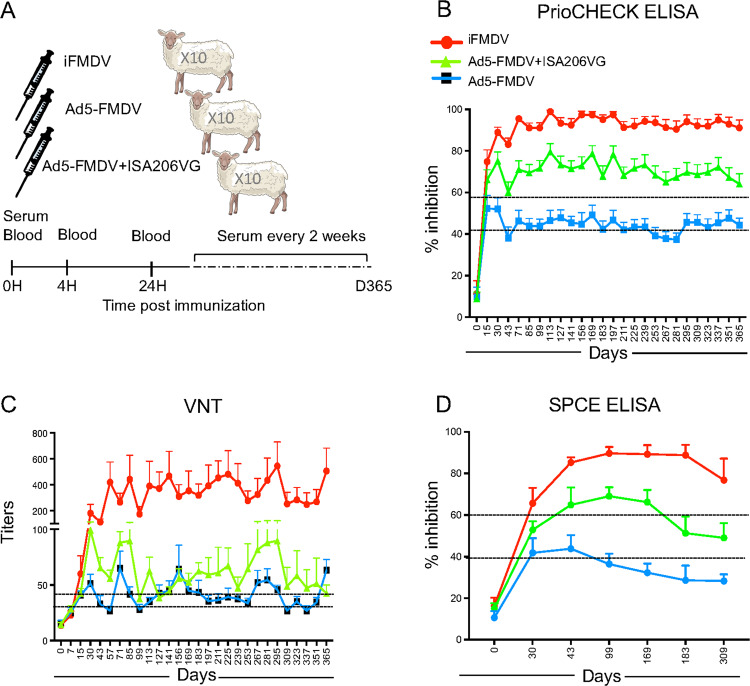
Humoral responses induced by iFMDV, Ad5-FMDV, and Ad5-FMDV+ISA206VG vaccine in sheep. **a** Experimental design: Three groups of 10 sheep each were immunized with iFDMV (intramuscular route), or Ad5-FMDV with or without ISA206VG (half intramuscular and half subcutaneous route). Blood was collected on PAXgene® Blood RNA tubes at T0H, T4H, and T24H and processed to RNA-seq. Sera were collected at T0H and every 2 weeks for 365 days. **b** The mean percent inhibition values provided by the PrioCHECK ELISA in each group at each time point are plotted over time (365 days), and the standard errors of the mean are shown above the mean (10 sheep per group). Differences between the areas under the curve over time of the three groups are statistically significant (*p* < 0.001, Wilcoxon test). The doubtful and positivity thresholds (42% and 58% respectively) are shown with a dashed line. **c** The mean VNT titer per group are plotted over time and the s.e.m. are shown above the mean. Differences between the areas under the curve of the iFMDV group and the other groups are statistically significant (*p* < 0.005, Wilcoxon test). The differences between the areas under the curve of the Ad5-FMDV and Ad5-FMDV+ISA206VG groups are not significant. The doubtful and positivity thresholds (VNT titers of 32 and 45 respectively) are shown with a dashed line. **d** The mean percent inhibition values provided by the SPCE test in each group at each time point are plotted over time (365 days), and the s.e.m. are shown above the mean. Differences between the areas under the curve over time of the three groups are statistically significant (*p* < 0.005, Wilcoxon test). The doubtful and positivity thresholds (40% and 60% respectively) are shown with a dashed line.

We aimed at comparing the serological responses induced by iFMDV and Ad5-FMDV in sheep as well as at evaluating the benefit of formulating Ad5-FMDV with Montanide ISA206VG, a mineral water-in-oil-in-water-based adjuvant previously shown to be efficient and well tolerated in sheep.^21^ Three groups of 10 sheep each were immunized in parallel with a single injection. Their serological responses were monitored every 2 weeks during a year by using the commercial PrioCHECK blocking ELISA kit and by conducting VNT assays. At selected time points, sera were also analyzed with an in-house SPCE assay, as a confirmation test (Fig. 1). By comparing the serological values per group over time, we observed that sheep from the iFMDV group displayed the highest responses with values well above the positive thresholds in all assays (see Methods), whereas sheep in the Ad5-FMDV group developed weak responses, mainly in the doubtful ranges (Fig. 1b–d). The use of ISA206VG appeared to promote the Ab responses (Fig. 1b–d). Overall the Ab responses reached a plateau at about 60 days post-vaccination in the three vaccinated groups and were maintained for at least a year.

**Fig. 2:**
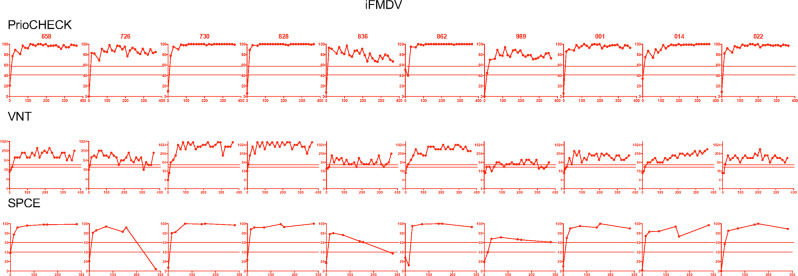
Humoral responses induced by the iFMDV vaccine in sheep individuals. The percent inhibition values obtained with the PrioCHECK and SPCE tests (linear scale) and the VNT titers (inverse of dilutions, log2 scale) are reported over time (D0 to D365) for each sheep, identified with its number. The doubtful and positive thresholds are indicated by dashed lines as in Fig. 1. Sheep were qualified positive responders and represented in red plots when the test values were considered positive in at least 30% of the time points, which is the case in all sheep vaccinated with iFMDV.

**Fig. 3:**
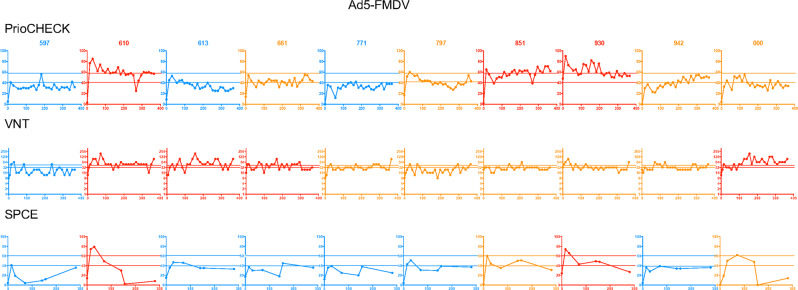
Humoral responses induced by the Ad5-FMDV vaccine in sheep individuals. The percent inhibition values obtained with the PrioCHECK and SPCE tests (linear scale) and the VNT titers (inverse of dilutions, log2 scale) are reported over time (D0 to D365) for each sheep, identified with its number. The doubtful and positive thresholds are indicated by dashed lines as in Fig. 1. Sheep were qualified positive responders and represented in red plots when the test values were considered positive in at least 30% of the time points. Sheep were qualified negative responders and represented in blue plots when the test values were considered negative (below the doubtful threshold) in at least 80% of the time points. They were qualified ambiguous in all other cases and represented in orange.

**Fig. 4:**
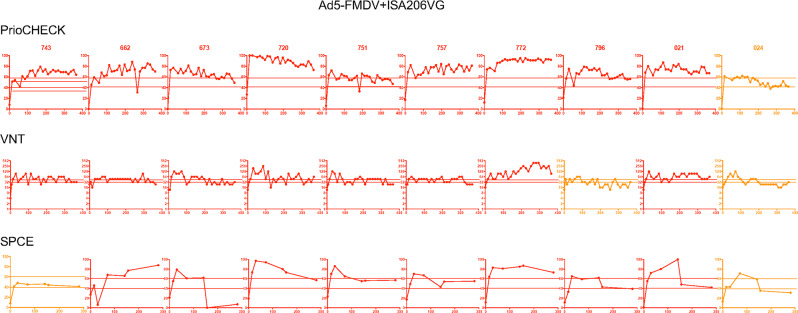
Humoral responses induced by Ad5-FMDV+ISA206VG vaccine in sheep individuals. The percent inhibition values obtained with the PrioCHECK and SPCE tests and the VNT titers (inverse of dilutions, log2 scale) are reported over time (D0 to D365) for each sheep, identified with its number. The doubtful and positive thresholds are indicated by dashed lines as in Fig. 1. Sheep were qualified positive responders and represented in red plots when the test values were considered positive in at least 30% of the time points. Sheep were qualified negative responders and represented in blue plots when the test values were considered negative (below the doubtful threshold) in at least 80% of the time points. They were qualified ambiguous in all other cases and represented in orange.

With the goal to capture the heterogeneity of responses induced by each vaccine, we illustrated the individual responses detected in each group with the three assays over time in Figs 2–4. In order to take into account the positive response thresholds and the variation of responses between time points, we arbitrarily qualified animals as positive responders when individual responses were positive in 30% of the analyzed time points, and non-responders when negative responses were found in 80% of the time points. In the iFMDV group, all sheep presented a serological response above the positive threshold at most time points with all three tests (Fig. 2). Notably, saturating values were rapidly reached with the PrioCHECK assay. Although all sheep were considered as positive responders based on the criteria presented above, heterogeneous levels of VNT titers were nevertheless encountered among sheep, with two higher responders (730 and 828) and two lower responders, although positive at most time points (836 and 989).

In the Ad5-FMDV group, responses were much more ambiguous (Fig. 3). With the PrioCHECK assay, three sheep were positive responders (610, 851, and 930), whereas three sheep were regularly found negative (597, 613, and 771). The status of the other sheep was unclear (661, 797, 942, and 000). With regard to the VNT titers, some sheep were qualified as responders based on our criteria (610, 613, 661, and 000). However this VNT finding did not match with the PrioCHECK’s or with the SPCE’s results (Fig. 3). In particular, three sheep showed positive VNT titers after 100 days and not before, a delay which is unexpected in primary immune responses. The PrioCHECK assay relies on a competition with a monoclonal Ab; therefore, a discrepancy with VNT results could be explained by differences in antigenic site recognition. The SPCE results, which rely on the competition with a polyclonal serum presumably interacting with several antigenic sites, nevertheless did not match the VNT results (sheep 613 and 661 in particular, Fig. 3).

We then evaluated the congruency between the three serological tests, by establishing the frequency at which a positivity given by one test is confirmed at least by another test (Table 1). In the Ad5-FMDV group, the VNT positive results were only confirmed by another test in 33 instances among 50. As the VNT titers were generally low in the Ad5-FMDV group and merely around the positivity threshold, we assume that the variability in antibody titers which is inherent to tests using cell cultures may affect whether a serum is classified as positive, doubtful, or negative at a certain time point. Alternatively, there may be non-specific viral inhibition in the virus neutralization test due to sheep immune responses to an irrelevant stimulus in the sera. Finally, in the Ad5-FMDV+ISA206VG group, most sheep were positive responders based on all tests (Fig. 4), and there was a better congruency for the VNT than in the Ad5-FMDV group (Table 1). Furthermore, there was a good correlation (Pearson *R* = 0.70, *p* = 0.0065) between the VNT and the PrioCHECK results (Supplementary Fig. 1). The levels reached by the VNT titers were most often beyond the threshold considered to be protective.^22,23^ Notable heterogeneity of responses was also observed in that group (Fig. 4).

**Table 1:** Positive and doubtful results confirmed with at least another serological test.

Vaccines	Serological test (5 timing per group)		
	PrioCHECK	SPCE	VNT
iFMDV	50 (50)/50^a^	48 (48)/50	49 (50)/50
Ad5-FMDV	45 (45)/50	46 (39)/50	33 (36)/50
Ad5-FMDV+ISA206VG	47 (50)/50	34 (44)/50	43 (49)/50

^a^Frequency of positivity confirmed with another serological test, and, in brackets, frequency of doubtful results confirmed with another serological test. A total of 50 sera were tested (10 per vaccine, 5 timing)

Overall the serological analyses show that the Ab response magnitude over time is much higher with the iFMDV than with the Ad5-FMDV vaccine in sheep, and that it is substantially increased by adding ISA206VG to Ad5-FMDV. Heterogeneous responses among sheep were obtained with the three regimens.

### iFMDV, ad5-FMDV and ad5-FMDV+ISA206VG induce functional transcriptional signatures in blood cells early after vaccination

Genome-wide transcriptional profiling early after vaccination in human (see ref. ^24^ for a review), and recently in sheep,^20^ has provided insight into the cellular and molecular mechanisms that underlie the adaptive antibody and T cell responses. In order to analyze the early gene expression alterations induced by the three vaccine regimens, blood cells were collected at T0H, T4H, and T24H post-vaccination to perform RNA-seq as the transcriptomic responses to adenovirus-based vaccine had been shown to peak at T24H in humans^25^ and this timing was also optimal for an adjuvanted protein vaccine^26^ and inactivated vaccines.^27^ After gene assignments of the reads onto the ovine genome with updated UTR boundaries (see Methods), a PCA of the gene counts (Supplementary Fig. 2) revealed that for all three vaccines, the T0H and T4H are not well separated and globally oppose to the T24H samples, without outliers. The number of differentially expressed genes (DEGs, Benjamini-Hochberg adjusted *p* value below 0.01) between T4H and T0H was quite small with all vaccines (Table 2, Supplementary Data Set 1), in agreement with the PCA results. However larger numbers of DEGs were identified between T24H and T0H, i.e. 352 in the iFMDV group, 3414 in the Ad5-FMDV group, and 1233 in the Ad5-MV+ISA206VG group.

**Table 2:** Number of DEGs in the three vaccinated groups.

	FMDVi	Ad5-FMDV	Ad5-FMDV+ISA206VG
T0H vs. T4H	29	173	134
T0H vs. T24	352	3414	1233

**Fig. 5:**
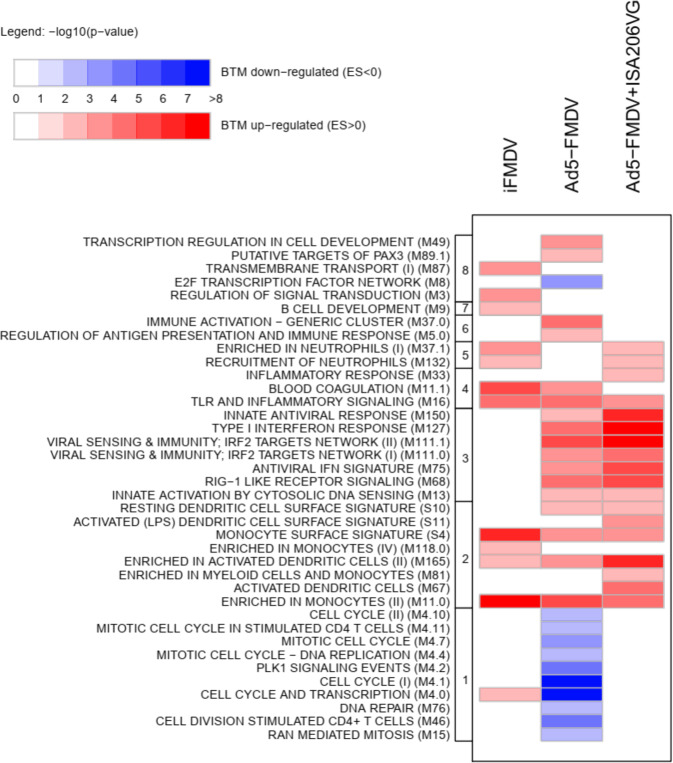
Modulated BTMs in sheep blood at T24H vs. T0H. Enriched BTMs were identified from the ranked list of the differentially expressed genes between T24H and T0H in sheep blood post-vaccination. The enrichment *p* values are represented by graded red (upregulated BTMs) and by graded blue (downmodulated BTMs). BTMs are grouped in functional families (cell cycle (1), mononuclear phagocytes (2), antiviral response (3), inflammation (4), neutrophils (5), immune response (6), B cell (7), cell metabolism (8)).The lists of the enriched BMTs and their statistical values are provided in Supplementary Data Set 2 (iFMDV), Supplementary Data Set 3 (Ad5-FMDV), Supplementary Data Set 4 (Ad5-FMDV+ISA206VG).

We next identified functional gene modules altered by the three vaccines at T24H. We identified enriched BTMs in the DEG list as described in ref. ^16^ and used recently in vaccine response studies in human blood^18,17,26,28^ as well as in sheep blood.^20^ The 334 BTMs were initially constructed from publicly available microarray data specific to human blood; they include cell-type-specific modules, allowing to point a cell type likely to be implicated in the gene expression modulation and we translated them into sheep BTMs (see Methods). The lists of the enriched BTMs induced by the three vaccines are provided in Supplementary Data Set 2 (iFMDV), 3 (Ad5-FMDV), 4 (Ad5-FMDV+ISA206V). The gene expression modulated between T4H and T0H did not retrieve interpretable functions. Interestingly at T24H, we retrieved several modules which were shared between the three vaccines (Fig. 5, Supplementary Data Set 2–4) and include upregulated BTMs related to monocytes (M11.0, S4), dendritic cells (M165), and to inflammation (M16). The BTMs restricted to the two Ad5-FMDV vaccines are related to responses to viruses and to interferon signaling (M111.1, M127, M68, M75, M111.0, M150, M13). Strikingly, Ad5-FMDV administration induced a strong systemic inhibition of many cell cycle-related modules (M4.0, M4.1, M4.2, M4.4, M4.7, M4.10, M4.11, M15, M46, M76) whereas, on the opposite, iFMDV induced the activation of one of these modules (M4.0). Addition of ISA206VG to Ad5-FMDV suppressed the viral effect on cell cycle activity and induced additional BTMs related to dendritic cells (M67, S11), monocytes (M81), and inflammation (M33).

Altogether this functional analysis indicates that the three vaccines induce several common innate responses at T24H which are related to mononuclear phagocyte and inflammatory activities. In contrast to the inactivated viral vaccine, the adenovirus-based vaccines induced systemic antiviral responses. Importantly, the addition of the ISA206VG adjuvant to Ad5-FMDV focused the blood response towards immunity-related functions.

### iFMDV, Ad5-FMDV, and Ad5-FMDV+ISA206VG induce early gene responses associated with the Ab response magnitude

**Fig. 6:**
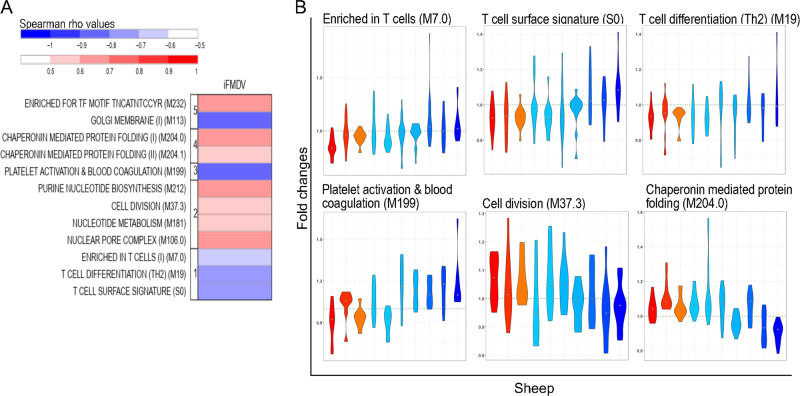
Transcriptomic signatures of the Ab response magnitude induced by the iFMDV vaccine. A PLS algorithm was used to retrieve the genes with fold changes (T24H vs. T0H) related to the VNT area under the curve. The ranked gene list of the first PLS component was processed through a GSEA to identify enriched BTMs (nominal *p* value of the GSEA < 0.05, FDR < 0.25). The gene fold changes contributing to each BTM were computed into a single activity score and a Spearman correlation test with VNT was performed across the 10 sheep (*p* < 0.05). **a** The BTMs positively correlating with the Ab response magnitude are in graded red according to the Spearman rho coefficient, and the negatively correlating ones are in graded blue. The BTMs are grouped in functional families (T cell (1), cell cycle (2), inflammation (3), Chaperonin-mediated folding (4), metabolism (5)). **b** The fold changes of genes contributing to the selected BTM (*y*-axis) are represented in a violin plot for each sheep which are ordered (*x*-axis) according to their VNT area under the curve values (sheep 828, 730, 862,014, 001, 658, 022, 726, 836, 989). Each sheep was attributed a color within a progressive gradient with red in the maximal responder and blue in the lowest responder (see Fig. 2). Six modulated BTMs, selected for their biological representativity, are illustrated. The list of the selected BMTs and their statistical values is provided in Supplementary Data Set 5 and the list of genes contributing to selected BMTs and their fold changes in high and low responders is shown in Supplementary Fig. 3.

In order to identify the innate responses which may explain the heterogeneity of the Ab responses, we evaluated the correlations between the magnitude of the Ab responses and the modulated blood transcriptome at T24H vs. T0H. On the basis of the serological analysis described above, in the iFMDV group, we selected the VNT values to perform the correlation: indeed PrioCHECK values were saturated in many instances (Fig. 2), the VNT positivity was almost always confirmed with another test (Table 1), and VNT titers are considered as correlates of protection.^23^ The area under the curve was calculated for each sheep over one year and used in a PLS analysis with the gene expression fold changes between T0H and T24H, in order to unravel their multivariate relationships with the Ab response. The genes from the PLS first component were ranked by their contribution and loaded on a GSEA using the sheep BTMs as gene sets (GSEA significance, *p* < 0.05, FDR < 0.25). The retrieved BTMs correlated with the VNT titer (Spearman, *p* < 0.05) are shown Fig. 6a and the list of the correlated BTMs with their statistical values is available in Supplementary Data Set 5. The expression of BTMs related to T cell responses (S0, M19, M7.0) as well as to an inflammation-type response (platelet activation, M199) at T24H after vaccination was negatively associated with the VNT titer magnitude, whereas the cell division (M37.3, M212, M181) and chaperonin-mediated protein folding (M204.0, M204.1) were positively associated. The gene fold changes contributing to six representative BTMs for each sheep are shown in Fig. 6b and the corresponding contributing gene lists (GSEA-core enrichment genes of the BTM limited to the top 25 genes, as ordered by the PLS) are presented in Supplementary Fig. 3. This representation shows that high Ab responders showed a global down-regulated expression of the genes of the T cell and platelet activation BTMs and an upregulated expression of the genes of the cell division and chaperonin-mediated protein folding BTMs. Therefore, as observed in other human and sheep systems vaccinology studies,^16,17,20,29^ we find that early downmodulation of T cell responses in blood is beneficial to later Ab responses.

**Fig. 7:**
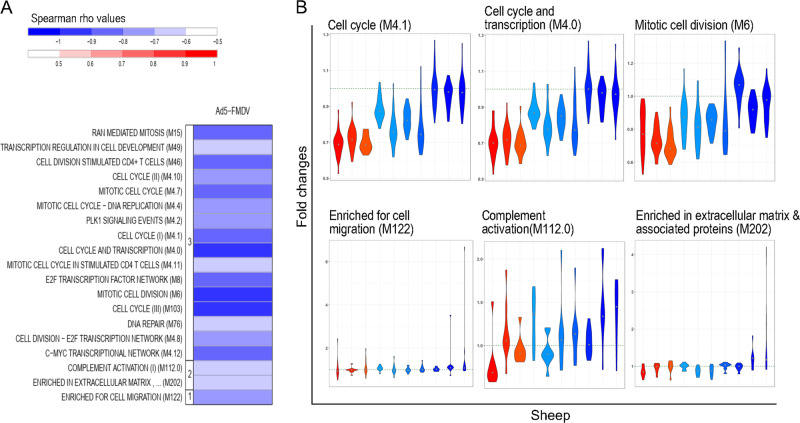
Transcriptomic signatures of the Ab response magnitude induced by the Ad5-FMDV vaccine. A PLS algorithm was used to retrieve the genes with fold changes (T24H vs. T0H) related to the PrioCHECK area under the curve. The ranked gene list of the first PLS component was processed through a GSEA to identify enriched BTMs (nominal *p* value of the GSEA < 0.05, FDR < 0.25). The gene fold changes contributing to each BTM were computed into a single activity score and a Spearman correlation test to VNT was performed across the 10 sheep (*p* < 0.05). **a** The BTMs negatively correlating with the Ab response are in graded blue. The BTMs are grouped in functional families (cell migration (1), inflammation (2), cell cycle (3)). **b** The fold changes of genes contributing to the selected BTM (*y*-axis) are represented in a violin plot for each sheep which are ordered (*x*-axis) according to their PrioCHECK area under the curve values (sheep 930, 610, 851, 797, 661, 942, 000, 613, 771, 597). Each sheep was attributed a color within a progressive gradient with red in the maximal responder and blue in the lowest responder (see Fig. 3). Six modulated BTMs, selected on their biological meaning, are illustrated. The list of the selected BMTs and their statistical values is provided in Supplementary Data Set 6 and the list of the genes contributing to the selected BMTs and their fold changes in high and low responders is shown in Supplementary Fig. 4.

**Fig. 8:**
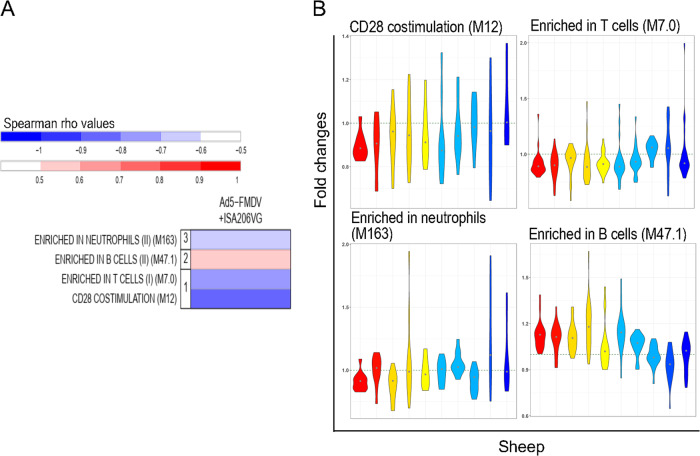
Transcriptomic signatures of the Ab response magnitude induced by the Ad5-FMDV+ISA206VG vaccine. A PLS algorithm was used to retrieve the genes with fold changes (T24H vs. T0H) related to the PrioCHECK area under the curve. The ranked gene list of the first PLS component was processed through a GSEA to identify enriched BTMs (nominal *p* value of the GSEA < 0.05, *p* < 0.25). The gene fold changes contributing to each BTM were computed into a single activity score and a Spearman correlation test to VNT was performed across the 10 sheep (*p* < 0.05). **a** The BTMs positively correlating with the Ab response magnitude are in graded red according to the Spearman rho coefficient, and the negatively correlating ones are in graded blue. The BTMs are grouped in functional families (T cell (1), B cell (2), neutrophils (3)). **b** The fold changes of genes contributing to the selected BTM (*y*-axis) are represented in a violin plot for each sheep which are ordered (*x*-axis) according to their PrioCHECK area under the curve values (720, 772, 021, 757, 662, 673, 743, 796, 751, 024). Each sheep was attributed a color, red in the maximal responders, yellow in intermediate responders, and blue in the lower responders (see Fig. 4). Four modulated BTMs, selected on their biological meaning and top positions, are illustrated. The list of the enriched BMTs and their statistical value is provided in Supplementary Data Set 7 and the list of the genes contributing to the selected BMTs and their fold changes in high and low responders is shown in Supplementary Fig. 5.

In the case of the Ad5-FMDV vaccine, the VNT values were often not confirmed with another test (Table 1) whereas the PrioCHECK values were most often confirmed and were not saturated (Fig. 3). We therefore used the PrioCHECK values to perform the correlations between the serological response and the transcriptomic data, both for the Ad5-FMDV and the Ad5-FMDV-ISA206VG groups, using the area under the curve over a year (see Methods, Supplementary Data Set 6 and 7 for the list of correlated BTMs). In the Ad5-FMDV group, most enriched functions were negatively correlated with the Ab response magnitude, namely many cell cycle modules (M4.0, M4.1, M103, M6, M4.7, M15, M46, M4.4, M4.8, M4.10, M76, M4.11), complement activation (M112.0) and enriched in extracellular matrix (M202) (Fig. 7, Supplementary Fig. 4). In the Ad5-FMDV+ISA206VG group, the correlated functions were more related to immune responses than in the Ad5-FMDV group. The enriched in B cell module (M47.1) shows a positive correlation with the Ab response with an increased expression of the contributing genes in the high Ab responders (Fig. 8, Supplementary Fig. 5). Notably, modules related to neutrophils and T cell activities presented a negative correlation with the Ab response (Fig. 8). Therefore, like in the iFMDV group, reduction of the T cell transcription activity has a positive impact on the Ab response elicited by Ad-FMDV in the presence of adjuvant, a feature not observed in absence of adjuvant.

In summary, most functions activated in blood by the three vaccines at T24H, such as monocyte, dendritic cell, and inflammatory responses, as well as the antiviral responses induced by the two adenovirus-based vaccines, are distinct from the functions associated with Ab response intensity. Interestingly, downmodulated T cell activities at T24H are associated with high responses to iFMDV and Ad-FMDV/ISA206, which are the two most potent vaccines in our study. The addition of the ISA206VG adjuvant to Ad5-FMDV focused the gene responses to immunity-related functions and also led to a positive association between high Ab response and early B cell activity in blood. Cell cycle activities at T24H had an opposite effect depending on the vaccine type, being positively correlated with Ab responses to iFMDV and negatively to non-adjuvanted Ad5-FMDV.

## Discussion

We showed that iFMVD, Ad5-FMDV, and Ad5-FMDV+ISA206 display variable potencies at inducing Ab responses in sheep. In order to explore the mechanisms directing the magnitude of the humoral responses that were heterogeneous in each vaccinated group, we identified early immune correlates in the altered transcriptomic modules of total blood cells 24 h after vaccination. Notably, whereas the transcriptomic signatures underpinning the polyfunctional CD4^+^ and CD8^+^ T cell responses have been documented with adenovirus-vectored vaccines in humans,^18,25,30^ the signatures correlated with antibody responses induced by adenovirus-vectored vaccines have not yet been identified, to the best or our knowledge. We propose that the correlates that we identified correspond to the early responses that dominantly impact on the different FMDV vaccine potencies at inducing Ab responses.

The experimental conditions that we used were particularly suitable to dissect immune response mechanisms with a systems vaccinology approach. Firstly, sheep, like other large outbred mammals such as cattle, pigs, and non-human primates, display variable levels of immune-competence between individuals, allowing correlation studies between parameters. At the difference with humans, these large mammals are raised in the protected environment of animal facilities, where biosafety measures are applied and food and exposome are controlled; therefore, the immunogenicity of vaccines is expected to be less influenced by external factors. Regarding pre-existing immunity in this study, sheep were non-immune to FMDV, which was not the case in many systems vaccinology studies conducted on responses to influenza vaccines.^16,17,27,31,32^ Importantly, previous studies have shown that ruminants do not have cross-reactive antibodies against human Ad5,^33^ which could have biased the results like found in humans.^25^ Besides pre-existing immunity and environment, conclusions from systems vaccinology studies can also be strongly affected by the age of the individuals,^32^ the use of PBMCs, or whole-blood cells, the arm of the considered adaptive immune response (humoral or cellular), and the timing of the transcriptome. Day 1, 3, and 7 post-vaccination are the most considered timings although they are often not all included.^15,25,27,31^ Therefore, comparative studies across vaccines conducted in parallel offer an important robustness, such as in ref. ^16^ and in the present study. However, although we worked under controlled conditions and focused on the Ab response, we used different read-outs, i.e. PrioCHECK ELISA for the Ad5-FMDV groups and VNT results for the iFMDV group. This choice was made to provide the most robust adaptive serological response overtime into the correlation study in each group. Notably in the Ad5-FMDV+ISA206VG group, the VNT and PrioCHECK ELISA values were well correlated (Supplementary Fig. 1).

The transcriptomic signatures analyzed in this study were derived from the BTMs established by Li et al.,^16^ an approach which has more discriminative power than classical canonical pathways analyses, and well adapted to blood cells and innate immunity response analyses. An important feature of BTMs is that the co-regulated genes are assigned to a given subset such as T cells, monocytes, and B cells. The modulated activity of cell-type-associated BTMs could be the result of modulation of transcription or of cell subset trafficking between blood and other compartments such as lymph nodes, which are the site of antigen presentation. Transcriptomic module analyses were conducted upon Ad5 immunization in mice,^30^ comparing the responses in the blood and lymph node compartments. Modules related to interferon responses were concordant in both places, but the ones related to inflammation and monocytes were discordant and indicated an accumulation of monocytes in blood and a depletion in lymph node upon Ad5 administration, a finding also confirmed in an independent study.^25^

We observed that our three different vaccines induced several similar functional activities in blood at T24H, i.e. monocyte, dendritic cell, and inflammatory responses, which are not associated with these vaccines’ efficacy at inducing high Ab responses. Similar systemic responses induced by different vaccines and unrelated to their efficiency have also been reported in other systems vaccinology studies when several vaccines were compared,^16,18^ and may even represent adverse effects of the vaccines,^32^ such as systemic inflammatory responses. Notably, iFMDV induces a lower number of DEGs in blood compared to the Ad5-FMDV regimens, and yet it is the most potent vaccine to induce Ab responses. In the case of Ad5-FMDV and Ad5-FMDV+ISA206VG, the early activation of the antiviral and interferon responses is expected to occur from Ad5 sensing through STING,^30^ RIG-1 like receptors,^34,35^ TLR,^36^ and possibly other pathogen-related receptor pathways. Interestingly, interferon responses were detrimental to recombinant human Ad5 and Chimpanzee Adenovirus 3 capacities to induce CD8^+^ T cells against SIV-encoded antigens, by decreasing the transgene expression.^30^ Finally we observed that the adjunction of the ISA206VG adjuvant focused the Ad5-FMDV vaccine response to immunity-related innate responses. The ISA206VG may act through avoiding a systemic dispersion of the Ad5-FMDV and providing additional intrinsic innate signaling as reported for the emulsion-type MF59 adjuvant.^37^

A striking finding obtained with the iFMDV and the Ad5-FMDV+ISA206VG vaccines is the negative correlation between the T cell modules and the Ab responses. Negative correlations related to T cell activities have been found in blood at T24H in human patients immunized with inactivated Flu vaccines.^17,16,29,31^ Such a negative correlation has also been identified at T72H in sheep post-immunization with an inactivated FMDV vaccine adjuvanted with TLR4 and 7 in liposomes^20^ and in human patients vaccinated with a polysaccharide vaccine against *Neisseria meningitidis*.^16^ Collectively, these findings suggest that a decrease of T cell activities in blood impacts positively on the Ab response induced by different vaccine types. We propose the hypothesis that the rapid and strong mobilization of T-cells to lymph nodes, reflected by a decrease in T cell BTM activity in blood, promotes the induction of humoral immunity.

B cell and cell cycle activities were found associated with Ab responses in a vaccine-dependent manner. A positive correlation between B cell activity at T24H and Ab responses was obtained only with the Ad5-FMDV+ISA206VG vaccine in this study. Such a positive association at T24H has also been observed in the case of the response to the AS01-adjuvanted circumsporozoite protein of *Plasmodium falciparum* in human patients.^18^ However in three vaccination trials with inactivated influenza, a negative correlation was found at T24H followed by a positive one at day 7.^29^ Regarding cell cycle BTMs, contrasted results were obtained in our study, as cell cycle activity was positively correlated in the case of the iFMDV and negatively correlated in the case of the Ad5-FMDV vaccine. Similarly in human patients, cell cycle activities at T24H were positively associated with Ab responses to adjuvanted influenza vaccine but negatively in response to non-adjuvanted influenza vaccine and this association was reversed at later time points.^17^ Therefore early cell cycle activities in blood are complexly related to immunogenicity, and probably depend on the vaccine-specific temporal effects on cell proliferation and migration between blood and lymph node compartments.

In total, our study demonstrates the clearly higher capacity of iFMDV to induce Ab responses compared to Ad5-FMDV in sheep, and shows that adjunction of ISA206VG leads to improved responses, with VNT levels reaching threshold associated to protection,^22,23^ at least for 1 year. Early transcriptomic signatures associated with Ad5-FMDV and iFMDV vaccine efficacy, such as the T cell response modules, can be proposed as early biomarker candidates of vaccine efficacy.

## Methods

### Sheep studies, ethic, and authorizations

The contained use of the GMO Ad5-FMDV was authorized by the “Ministère de l'Enseignement Supérieur, de la Recherche et de l'Innovation” following opinion of the “Haut Conseil des Biotechnologies”. All experiments were conducted in accordance with the EU guidelines and the French regulations (DIRECTIVE 2010/63/EU, 2010; Code rural, 2018; Décret no. 2013-118, 2013), and complied to the recommandations of the “Charte nationale portant sur l’éthique en expérimentation animale” established by the “Comité National de Réflexion Ethique sur l’Expérimentation Animale” (CNREEA—Ministère de l'Enseignement Supérieur, de la Recherche et de l'Innovation—Ministère de l’Agriculture et de l’Alimentation).

The animal experiment was approved by the Comité d'Éthique en Expérimentation Animale Val de Loire (CEEA VdL, committee no. 19) under the number APAFIS#3198-2015121515515154 v3 and was conducted at the “Plate-Forme d’Infectiologie Expérimentale” (PFIE, INRA, Nouzilly, France, 10.15454/1.5535888072272498e12). The Ad5-FMDV groups were immunized in A-BSL2 containment and 2 weeks later, they were placed in A-BSL1 containment together with the group vaccinated with iFMDV.

### Vaccines

The inactivated FMDV vaccine (iFMDV) is a binary ethylene-imine-inactivated monovalent AFTOVAXPUR DOE vaccine containing the O1 Manisa serotype, with the payload corresponding to the registered 6PD50 (50% protective dose in cattle as described in *European Pharmacopoeia Monograph* 0063). The batch was manufactured, controlled, and released as described in the European public assessment report: https://www.ema.europa.eu/en/documents/assessment-report/aftovaxpur-doe-epar-public-assessment-report_en.pdf.

The recombinant replication-defective human adenovirus type 5 (Ad5-FMDV) is deleted of the E1/E3 genes and encodes the structural protein P1 and non-structural proteins 2A and 2B of the FMDV O1 Manisa serotype fused in frame with the non-structural proteins 3B (partial) and 3C of FMDV serotype A12; it has been originally described in ref. ^38^ The Ad5-FMDV vaccine was produced in an HEK-293 cell line which complementes for the human adenoviral E1, allowing viral replication. Viral titers were determined using a focus-forming unit (FFU) assay. Briefly ﻿HEK-293 cells were infected with various dilutions and FMDV antigen was detected using indirect fluorescence with the F1412SA anti-FMDV mAb^39^ (5 µg/ml) followed by a goat anti-mouse Cy3 conjugate (1:100 dilution; Jackson Immuno Research, Cat. No.115-165-004). FFU/ml was determined by counting positive foci and applying dilution factors. The batch used in the vaccination titrated 4.5 × 10^8^ FFU/ml. Ad5-FMDV is non-replicative in sheep cells. Production of FMDV antigens occurs from transduced ovine cells following injection of the vaccine in sheep.

### Immunization of sheep

Sheep (Prealpes du Sud strain, 6 month-old) were divided into three groups of 10 male sheep each (Fig. 1A). One group (iFMDV) received 1 ml of the benchmark FMDV type O1 Manisa MERIAL vaccine by the intramuscular route, one group (Ad5-FMDV) received 3.52 × 10^7^ FFU of Ad5-FMDV per animal, and one group (Ad5-FMDV + ISA206VG) received 3.52 × 10^7^ FFU of Ad5-FMDV emulsified with the water-in-oil-in-water Montanide ISA206VG adjuvant. Half dose of the adenovirus-based vaccines was inoculated subcutaneously and the other half intramuscularly (2 ml in total) as we used before in sheep with recombinant canine adenovirus.^40^ Montanide™ adjuvants and their components have been considered as safe by the Committee for Veterinary Medical Products (CVMP) for use in immunological products and are included as authorized substances in the annex of the European Council Regulation no. 470/2009 (previously 2377/90/EC) or included in already registered veterinary commercial products.

### Sample collections

Blood was collected in PAXgene® Blood RNA tubes (BD Biosciences) for subsequent RNA extraction just before vaccine administration (T0H), 4 h after (T4H) and 24 h after (T24H). For serum collection, blood was collected at T0, and every 2 weeks during 1 year (Fig. 1a).

### ELISA

Sera were analyzed with the commercial PrioCHECK® FMDV type O blocking ELISA test (ThermoFisher Scientific, Rockford, USA, designated as PrioCHECK in this paper), which relies on a specific monoclonal Ab whose binding is blocked by the specific antibodies that are present in the sera. Results within [42; 58%] inhibition were considered doubtful and clearly positive when superior or equal to 58% inhibition, based on the coefficient of variation validated by the FMDV French National Reference Laboratory. The percent inhibition values were plotted over time, the curves were smoothed in order to mathematically correct for experimental variations (R function smooth.spline with spar = 0.5), and the areas under the curve over time were calculated, taking 42 as the baseline (approxfun and integrate R functions).

At specific time points, the sera were also analyzed with an in-house solid phase competition ELISA (SPCE) test. Based on the OIE Manual guideline^3^, plates were coated with a polyclonal rabbit serum targeted to serotype O FMDV antigens (1:5000 dilution; FMD World Reference Laboratory, Pirbright, UK) for the capture of inactivated serotype O vaccine antigen, produced by Merial, Boehringer Ingelheim, France. Subsequently the binding of a specific polyclonal guinea pig serum (1:8000 dilution; FMD World Reference Laboratory, Pirbright, UK) was blocked by the specific Abs that are present in the tested sera. The attachment of guinea pig Abs was revealed by addition of polyclonal rabbit anti-guinea pig immunoglobulins conjugated to horseradish peroxidase (1:700 dilution; DakoCytomation, Denmark) and o-phenylenediamine dihydrochloride peroxidase substrate (Sigma-Aldrich, Germany). Results were expressed as percent inhibition of attachment of antibodies from guinea pig sera and considered doubtful when within [40; 60%] inhibition and clearly positive when superior or equal to 60% inhibition, according to validations by the FMDV French National Reference laboratory.

### Viral Neutralization Test assay

A Viral Neutralization Test (VNT) assay was conducted following the protocol of the OIE Manual [, 2014 #20], using homologous O1 Manisa virus. The virus titers and the titer of positive working control sera were charted, monitored, and compared to their predetermined values. The VNT titer for each serum was established as the inverse of the last serum dilution which neutralizes 50% of the wells. A serum with a VNT titer within [32; 45] was considered doubtful and a serum with a VNT titer of 45 and above was considered as clearly positive. The VNT titers were plotted over time, the curves were smoothed as described above, and the areas under the curve over time were calculated, taking 32 as the baseline (approxfun and integrate R functions).

### RNA extraction, quality check, and sequencing

RNA was extracted from the PAXgene® Blood RNA tubes using the PAXgene® Blood RNA kit and purified with the RNeasy MinElute Cleanup kit (Qiagen). A total of 90 RNA samples were prepared (30 sheep, 3 time points: T0H, T4H, T24H). The quality of the total RNA was assessed on an Agilent Bioanalyzer 2100, using RNA 6000 pico kit (Agilent Technologies). Directional RNA-Seq Libraries were constructed from 1 µg of total RNA using the TruSeq mRNA Stranded Library Prep Kit (Illumina), following the manufacturer’s instructions. The final libraries’ quality was assessed with an Agilent Bioanalyzer 2100, using an Agilent High Sensitivity DNA Kit. Libraries were pooled in equimolar proportions and sequenced in paired-end runs (51 nt forward–34 nt reverse) on an Illumina NextSeq500 instrument, using NextSeq 500 High Output 75 cycles kits. Demultiplexing has been done with bcl2fastq2 V2.2.18.12. Adapters were trimmed with Cutadapt1.12 and only reads longer than 10 pb were kept.

### Bioinformatic analyses

The Illumina sequencing produced in average 180 ± 29 millions of reads (R1 + R2) per sample (minimum: 123 millions; maximum: 294 millions). In total, 74.5% of the sequences could be aligned with tophat2 (v2.0.14; options: -N 2 –read-edit-dist 2 –b2-sensitive –no-coverage-search) on the ovine transcriptome (reference Ensembl 90 -Ovis aries 3.1). When trying to assign these alignments to genes using featureCounts (subreadds v1.5.2; options: -s 2 -p -C), we observed a loss of 45% of these alignments. Using Integrative Genome Viewer, we identified that a significant amount of the coverage loss was due to the poor annotation of 3′ and 5′ UTRs (only 20% of genes referenced in Ensembl 90 for Ovis aries have a defined 3′ UTR). Therefore, we interrogated Cufflinks (v2.2.1; options: –max-intron-length 300 –min-frags-per-transfrag 30) and Cuffmerge to propose a new gene model, based on our aligned libraries. Forward and reverse alignments were treated independently. The updates corresponding to lengthened 3′ or 5′ UTRs were selected. Among the genes expressed in our libraries (minimum 10 reads), 64% of their UTRs boundaries were updated. Using this new model, we succeeded to assign 77% of the aligned fragments to genes (in average, 56 ± 9 millions of fragments; minimum, 38 millions; maximum, 92 millions). The newly annotated genes are provided in Supplementary Data Set 8.

### Biostatistical analyses

The pipeline of the biostatistical analyses is provided in Supplementary Fig. 6. Gene counts were transformed using RLOG function of DESeq2 package (v1.18.1). Genes having a sum of less than 10 reads were excluded. The mixOmics (v6.3.1) multi-level Principal Component Analysis (PCA) algorithm was applied on the data in each vaccinated group to reveal a partition of the samples according to the time of blood collection.

For functional genomic analyses, we used the BTM proposed by Li et al. to analyze vaccine responses in human [Li, 2014, 24336226]. For each gene of the 3101 distinct genes referenced in the 346 human BTMs, we obtained a corresponding ovine gene for 2702 of them, either using gene symbol analogy or manually curated orthologies (87% of recovery). These gene identifiers were used to create a gmt file (https://software.broadinstitute.org/cancer/software/gsea/wiki/index.php/Data_formats#GMT:_Gene_Matrix_Transposed_file_format_.28.2A.gmt.29) which provides the ovine BTMs definitions (Supplementary Data Set 9).

In order to study the functional signatures of the gene expression changes between T0H and T24H in response to each vaccine (=vaccine response, Supplementary Fig. 6 for a descriptive workflow), we performed a differential analysis using the DESeq2 R package (v1.18.1).^41^ Genes having a Benjamini–Hochberg adjusted *p* value below 0.01 were selected and used as input to the BTM Fisher enrichment test proposed by Li et al.^16^ (Supplementary tutorial; Application Tutorial of Blood Transcription Modules—Part II: enrichment_test function). BTMs having an enrichment test *p* value below 0.01 were selected to produce the heatmap displayed in Fig. 5.

In order to identify the functional signatures of the gene expression correlating with the serological responses (=vaccine correlation; Supplementary Fig. 6 for a descriptive work flow), the gene expression fold change between T24H and T0H was computed for each gene of the 10 sheep per group. The mixOmics Partial Least Square (PLS) algorithm was used to unravel the multivariate relationship between the gene fold changes and the serological responses (areas under the curve). The genes were then ranked by their contribution to the PLS first component and they were used in a pre-ranked Gene Set Enrichment analysis to identify enriched BTMs (GSEA, http://software.broadinstitute.org/gsea). The enrichment statistic parameter was set to “classic”, a more conservative approach than the default value, as recommended in the GSEA user’s guide (pre-ranked analyses). BTMs having less than 10 genes were not considered (Min size = 10). BTMs showing a GSEA nominal *p* value below 0.05 and a FDR below 0.25 were selected. The activity scores of the enriched BTMs were computed using their gene expression fold changes between T24H and T0H, as proposed by Li et al.^16^ A Spearman correlation analysis between the BTM activity scores and the serological responses was conducted to identify the significantly correlated BTMs (Spearman correlation test, two-sided, *p* value <0.05). The correlated BTMs are displayed in Figs 6–8.

### Statistical analyses of the serological results

The areas under the curves of the three serological test results over time were calculated for each sheep and the values between the groups were compared using a Wilcoxon non-parametric test (two-sided).

### Reporting summary

Further information on research design is available in the Nature Research Reporting Summary linked to this article.

## Supplementary information

Reporting Summary

Supplementary Information

Supplementary data set 1

Supplementary data set 2

Supplementary data set 3

Supplementary data set 4

Supplementary data set 5

Supplementary data set 6

Supplementary data set 7

Supplementary data set 8

Supplementary data set 9

## Data Availability

All datasets, tools, and sheep samples developed by the authors are available to readers upon writing to the corresponding author. The RNA-seq data were deposited on Gene Expression Omnibus, NCBI series record GSE135609 (https://www.ncbi.nlm.nih.gov/geo/info/seq.html). The supplementary data sets are also available under 10.5281/zenodo.3545663.
