# Supplementary material for: The antibody response induced FMDV vaccines in sheep correlates with early transcriptomic responses in blood
Source: NPJ Vaccines. 2020 Jan 3;5:1. doi: 10.1038/s41541-019-0151-3 (PMC6941976; doi:10.1038/s41541-019-0151-3)
Supplement: Supplementary file 2 — Supplementary Information [file 41541_2019_151_MOESM2_ESM.pdf]

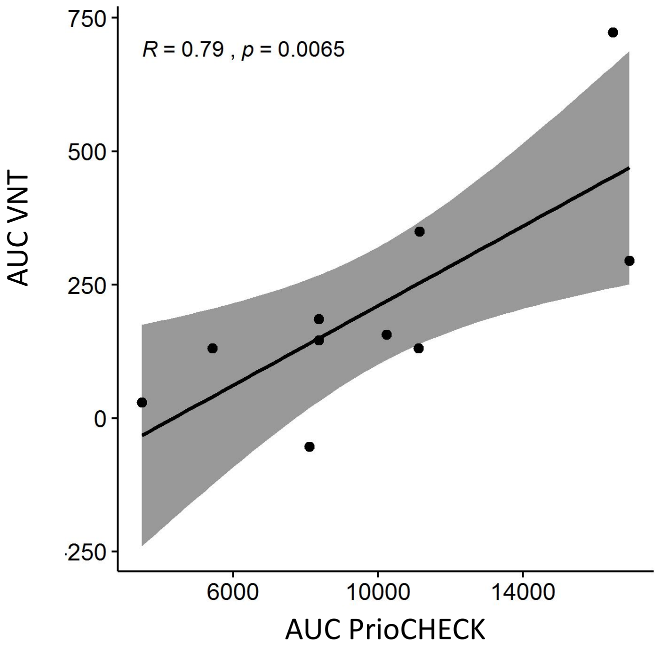


**Supplementary Figure 1.**The correlation between the area under the curve values (AUC) of the VNT titers and of the PrioCHECK values is illustrated (non parametric Spearman correlation ).

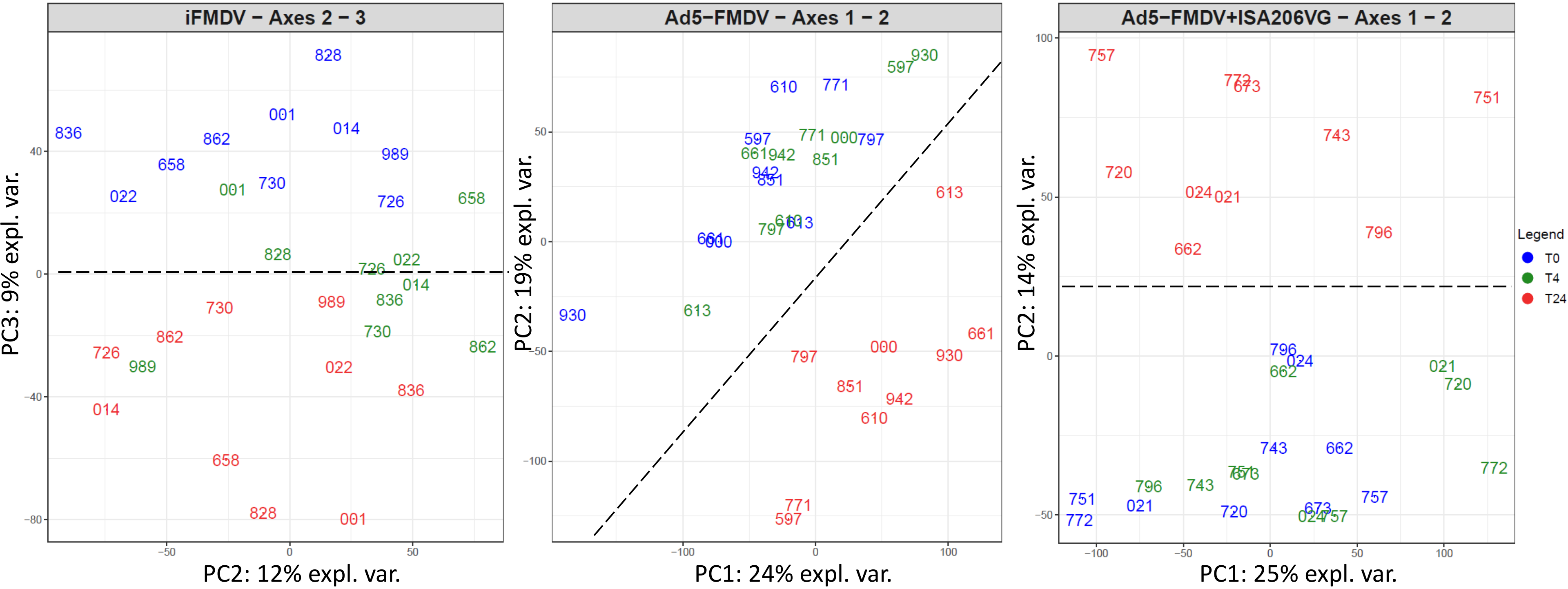


**Supplementary Figure 2.** Principal component analyses of the three groups of vaccination, taking into account the fact that each animal is sampled at three different time-points (multilevel decomposition of repeated measurements – Package mixOmics).
Ad5-FMDV group shows a clear separation between the T0H/T4H and T24H samples on the combination of axes 1 and 2.
Ad5-FMDV+ISA206VG group shows a similar separation on the axis 2 (14% of global variance).
T0H and T24H iFMDV samples are separated on axis 3 (9% of global variance), T4H samples showing an intermediate position.

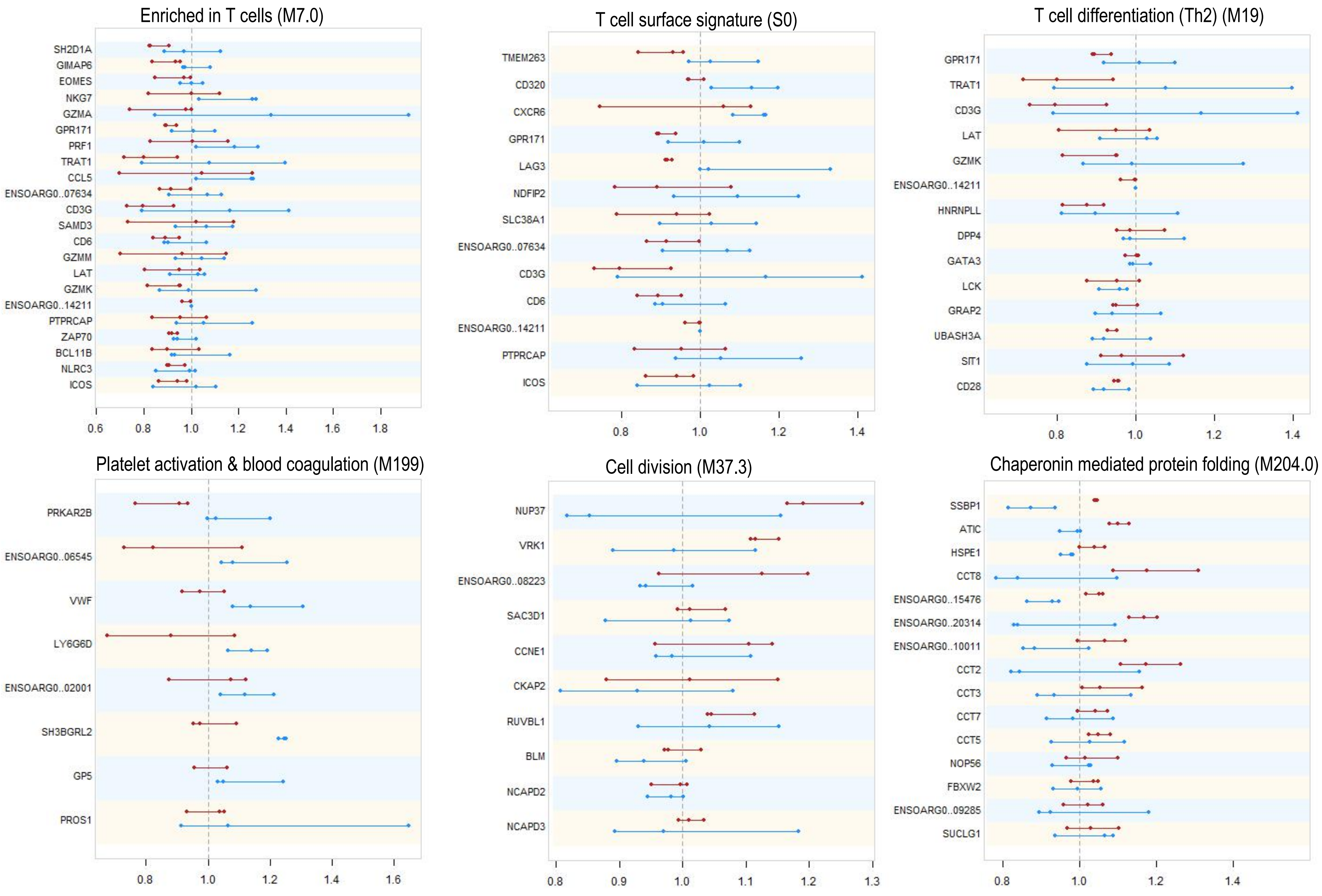


**Supplementary Figure 3** - Expression of the genes contributing to the selected BTMs correlated with the magnitude of the Ab responses induced by the iFMDV vaccine. The fold changes (T24H vs. T0H) of the genes of the BTMs selected in Figure 6B are shown for the 3 highest (red) and 3 lowest (blue) Ab responding sheep.

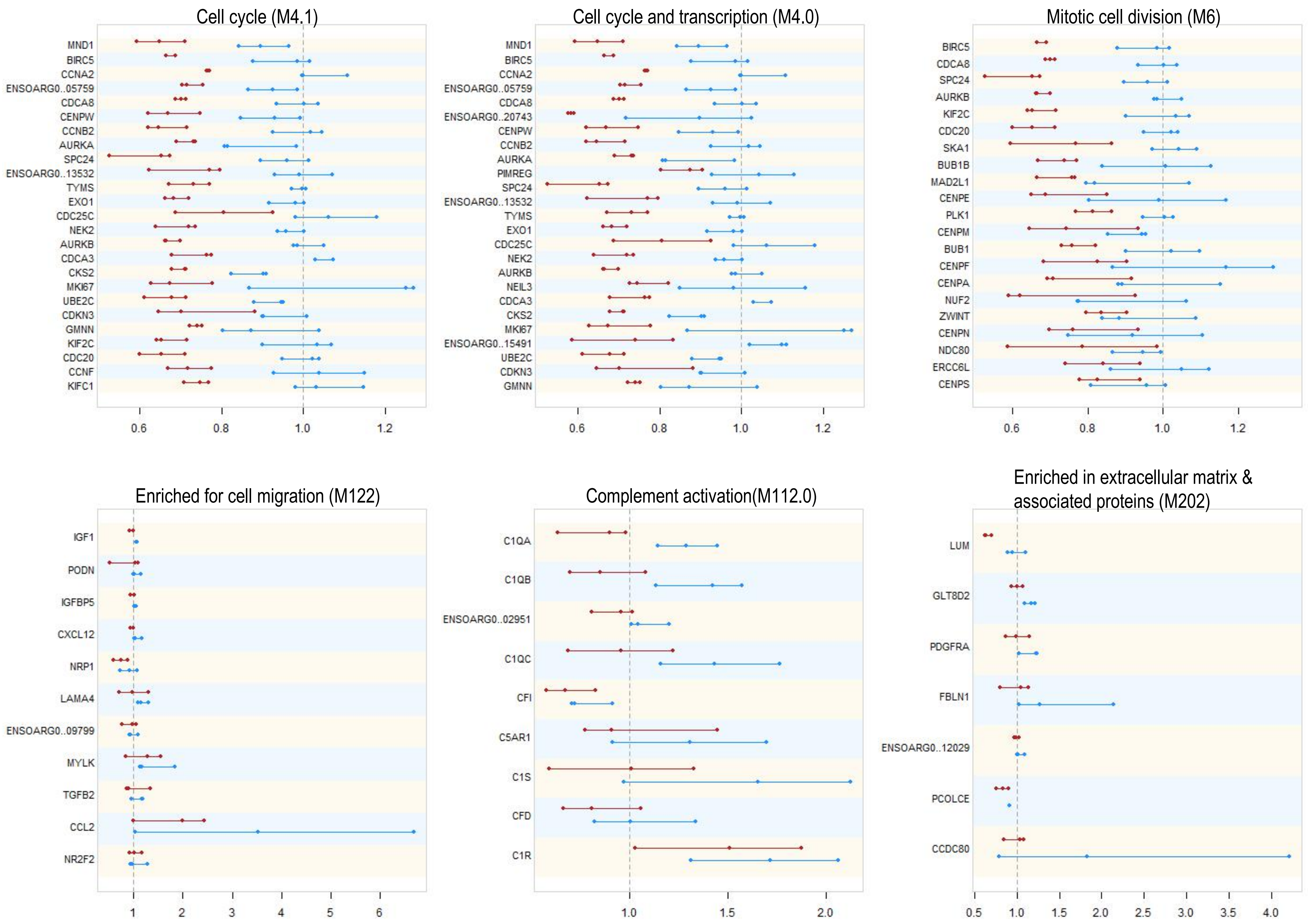


**Supplementary Figure 4.** Expression of the genes contributing to the selected BTMs correlated with the magnitude of the Ab responses induced by the Ad5-FMDV vaccine. The fold changes (T24H vs. T0H) of the genes of the BTMs selected in Figure 7B are shown for the 3 highest (red) and 3 lowest (blue) Ab responding sheep.

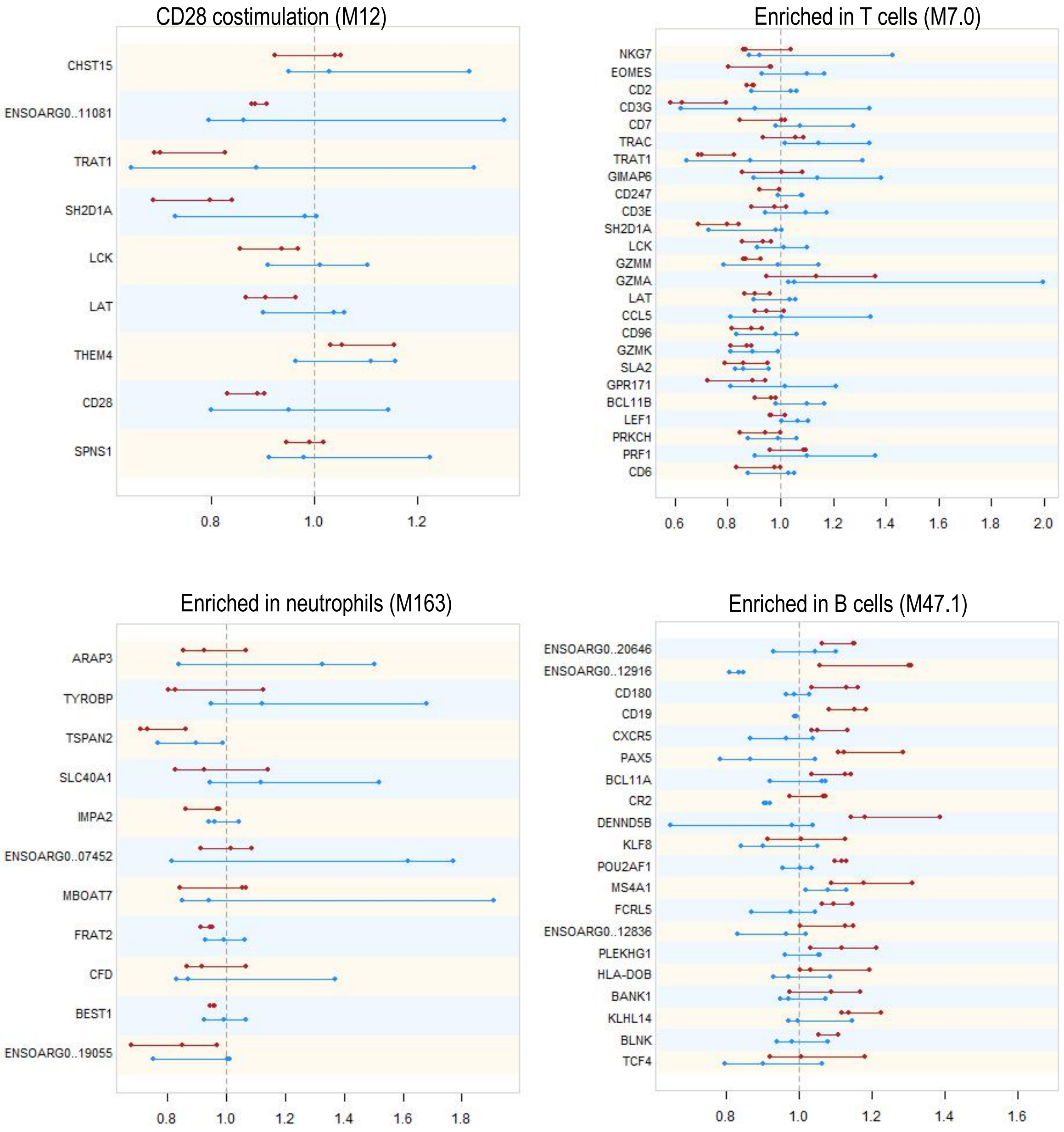


**Supplementary Figure 5.** Expression of the genes contributing to the selected BTMs correlated with the magnitude of the Ab responses induced by the Ad5-FMDV+ISA206VG vaccine. The fold changes (T24H vs. T0H) of the genes of the BTMs selected in Figure 8B are shown for the 3 highest (red) and 3 lowest (blue) Ab responding sheep.

# Analysis workflow

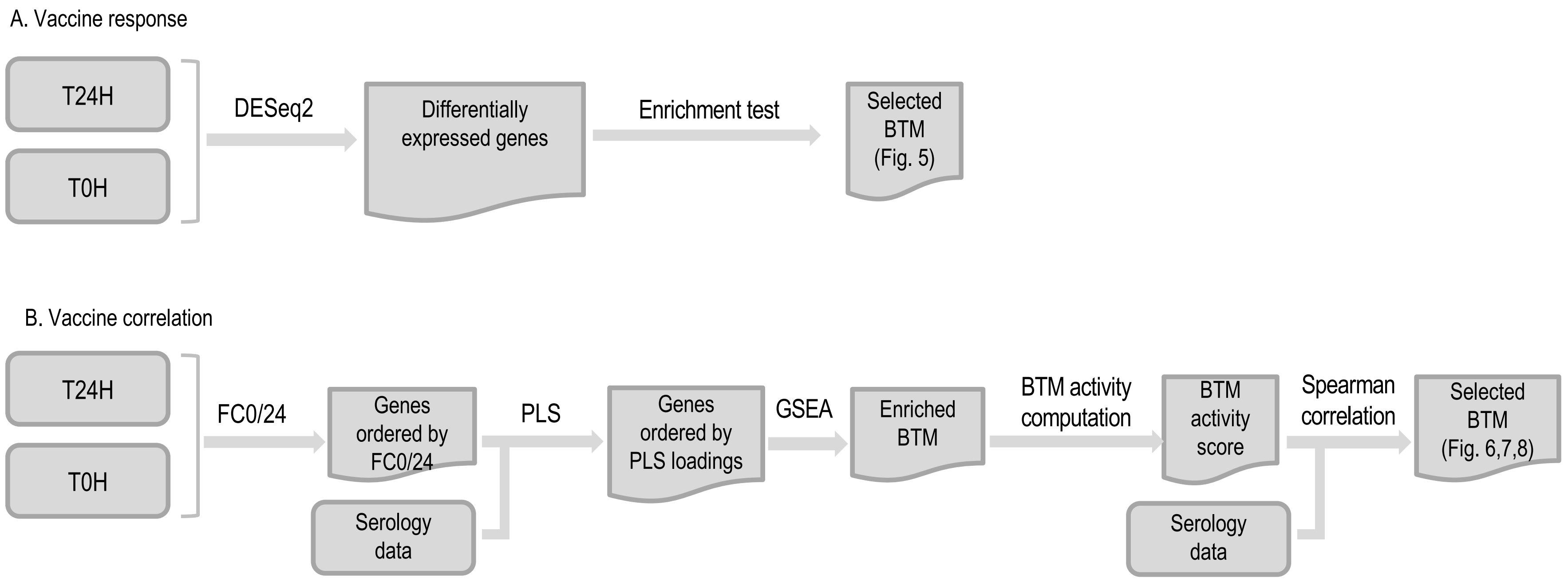


**Supplementary Figure 6.** Pipeline of the biostatistic analyses. **A.** The DESeq2 package was used to identify the differentially expressed genes (DEG) between T0H and T24H in each group, selecting the genes with adjusted p-values inferior to 0.01 (Benjamini-Hochberg procedure). A BTM enrichment test was used (DOI  10.1038/ni.2789, Supplementary tutorial : Application Tutorial of Blood Transcription Modules – Part II enrichment_test function) and BTMs with $p < 0.01$ were selected to generate Fig. 5.  **B.** The mixOmics Partial Least Square (PLS) algorithm was used to establish the relationship between the expression fold change between T0H and T24H (FC0/24) and the area under the curve of the serological response observed for each animal. Gene list ordered by their contribution to PLS first dimension was subjected to GSEA (http://software.broadinstitute.org/gsea) to identify the enriched BTMs. The enriched BTM activities were computed (DOI  10.1038/ni.2789, Supplementary tutorial : Application Tutorial of Blood Transcription Modules – Part II genetable_to_activityscores) and correlated to the antibody response using a Spearman test (p-value < 0.05) to generate  the final BTM selection represented in Fig. 6, 7 and 8.

**Legends of the Supplementary data sets**

Supplementary data set 1. DESEq2 results for the comparisons {T0h vs T4h} and {T0h vs T24h}.

Supplementary data set 2. List of the BTMs modulated in sheep blood at T24H vs. T0H in the iFMDV group. An BMT enrichment test was used from the Li et al paper (DOI 10.1038/ni.2789, Supplementary tutorial : Application Tutorial of Blood Transcription Modules – Part II enrichment_test function) and BTMs with $p < 0.01$ were selected.

Supplementary data set 3. List of the BTMs modulated in sheep blood at T24H vs. T0H in the Ad5-FMDV group. An BMT enrichment test was used from the Li et al paper (DOI 10.1038/ni.2789, Supplementary tutorial : Application Tutorial of Blood Transcription Modules – Part II enrichment_test function) and BTMs with $p < 0.01$ were selected.

Supplementary data set 4. List of the BTMs modulated in sheep blood at T24H vs. T0H in the Ad5-FMDV+ISA206VG group. An BMT enrichment test was used from the Li et al paper (DOI 10.1038/ni.2789, Supplementary tutorial : Application Tutorial of Blood Transcription Modules – Part II enrichment_test function) and BTMs with $p < 0.01$ were selected.

Supplementary data set 5. List of the BMTs correlated to the Ab response magnitude induced by the iFMDV vaccine. A relationship between the VNT area under the curve and the gene expression fold change (T24H vs. T0H) was established with a PLS algorithm. The ranked gene list of the first PLS component was processed through GSEA to identify enriched BTMs. The enriched BTM activities were computed and a Spearman correlation with the Ab response was calculated across sheep. BTMs with p-value < 0.05 were selected. The Spearman p-value and rho factor are provided for each BTM.

Supplementary data set 6. List of the BMTs correlated to the Ab response magnitude induced by the Ad5-FMDV vaccine. A relationship between the Priocheck values area under the curve and the gene expression fold change (T24H vs. T0H) was established with a PLS algorithm. The ranked gene list of the first PLS component was processed through GSEA to identify enriched BTMs. The enriched BTM activities were computed and a Spearman correlation with the Ab response was calculated across sheep. BTMs with p-value < 0.05 were selected. The Spearman p-value and rho factor are provided for each BTM.

Supplementary data set 7. List of the BMTs correlated to the Ab response magnitude induced by the Ad5-FMDV+ISA206VG vaccine. A relationship between the Priocheck values area under the curve and the gene expression fold change (T24H vs. T0H) was established with a PLS algorithm. The ranked gene list of the first PLS component was processed through a GSEA to identify the enriched BTMs. The enriched BTM activities were computed and a Spearman correlation with the Ab response was calculated across sheep. BTMs with p-value < 0.05 were selected. The Spearman p-value and rho factor are provided for each BTM.

Supplementary data set 8. List of the genomic coordinates of the 3' and 5' UTRs added to or modified from Ensembl90 Ovis aries transcriptome model. These updates have been inferred from the alignments of our 90 RNA-Seq libraries (3 sheep at 3 time points).

Supplementary data set 9. List of the Blood Transcriptomic modules adapted to sheep.
